# College of Physicians

**Published:** 1834-04-01

**Authors:** 


### COLLEGE OF PHYSICIANS.

The following note has been circulated, intended, we believe, as a reply to some observations recently made, on the College of Physicians, in the House of Peers :

1. The College of Physicians grants its license to practise within its jurisdiction to any man who has obtained the degree of Doctor of Physic in any university upon earth, after a residence of two years; and who, upon examination, proves his competency. This license enables him to receive the ordinary emoluments of physicians, makes him eligible to all public institutions, to become a lecturer and teacher at the great medical schools, and to hold the high offices of physician to their Majesties and the Royal Family.

2. The College of Physicians, however, having thus done justice to individuals seeking to practise as physicians within their jurisdiction, has thought itself at liberty to provide for the honour of the profession, by offering some inducement to those who are about to enter it, to obtain that previous education, which, in England, has always been esteemed the highest and the best: and the inducement consists in admitting, by preference, the Graduates of Oxford and Cambridge to the Fellowship of the College.

3. The Fellow of the College has no advantage whatever over the Licentiate, with reference to the public. He differs from the Licentiate only in belonging to the governing body of the College; and in this capacity he has certain duties to perform, and holds in rotation certain offices. Of these offices, those of President, four Censors, Treasurer, Registrar, and four Lecturers, receive emoluments. The emolument of the President is 25*l.* per annum; of each Censor, 20*l.* per annum; of the Treasurer, 25*l.* per annum; of the Registrar, 40*l.* per anuum; of the four Lecturers, the two juniors receive 10*l.* each; the third, 20*l.*; and the senior, 32*l.*

4. But, although the College has held forth the little honour and profit of the Fellowship as an inducemnt to those who become physicians to obtain the previous education of an English University, it has elected into the Fellowship from time to time, and continues to elect, eminent individuals from among the Licentiates. *Med. Gazette.*

